# Supplementary material for: Depression recurrence is accompanied by longer periods in default mode and more frequent attentional and reward processing dynamic brain‐states during resting‐state activity
Source: Hum Brain Mapp. 2023 Sep 6;44(17):5770–83. doi: 10.1002/hbm.26475 (PMC10619399; doi:10.1002/hbm.26475)
Supplement: Supplementary file 1 — Data S1. Supporting Information. [file HBM-44-5770-s001.pdf]

Supplementary Material

Supplementary Table S1. Fractional occupancy scores of states in partition k18 and associated statistics

	Recurring rrMDD-patients (i.e., relapse at FU; N=11)				Nonrecurring rrMDD-patients (i.e., no relapse at FU; N=17)		
state	Baseline mean ± std	Follow-up mean ± std	p	pFDR	Baseline mean ± std	Follow-up mean ± std	p
c1	0.35 ± 0.25	0.31 ± 0.22	0.282	0.391	0.36 ± 0.18	0.36 ± 0.14	0.485
c2	0.14 ± 0.09	0.13 ± 0.10	0.458	0.459	0.19 ± 0.09	0.16 ± 0.07	0.133
c3	0.11 ± 0.08	0.08 ± 0.05	0.142	0.391	0.07 ± 0.05	0.07 ± 0.06	0.324
c4	0.07 ± 0.06	0.03 ± 0.04	0.012	0.056	0.04 ± 0.02	0.04 ± 0.04	0.400
c5	0.06 ± 0.07	0.04 ± 0.03	0.197	0.391	0.03 ± 0.03	0.03 ± 0.03	0.411
c6	0.04 ± 0.04	0.04 ± 0.04	0.312	0.401	0.05 ± 0.04	0.03 ± 0.03	0.082
c7	0.03 ± 0.06	0.05 ± 0.12	0.231	0.391	0.01 ± 0.01	0.01 ± 0.02	0.193
c8	0.03 ± 0.03	0.05 ± 0.05	0.267	0.391	0.03 ± 0.04	0.05 ± 0.04	0.040
c9	0.03 ± 0.03	0.03 ± 0.04	0.459	0.459	0.03 ± 0.02	0.02 ± 0.01	0.045
c10	0.01 ± 0.02	0.05 ± 0.04	0.004	**0.039**	0.06 ± 0.06	0.07 ± 0.06	0.249
c11	0.03 ± 0.03	0.03 ± 0.03	0.431	0.459	0.03 ± 0.04	0.02 ± 0.03	0.205
c12	0.01 ± 0.01	0.05 ± 0.04	0.009	0.053	0.03 ± 0.02	0.04 ± 0.04	0.038
c13	0.01 ± 0.01	0.03 ± 0.04	0.155	0.391	0.03 ± 0.03	0.02 ± 0.02	0.176
c14	0.01 ± 0.02	0.02 ± 0.04	0.175	0.391	0.01 ± 0.02	0.02 ± 0.02	0.202
c15	0.01 ± 0.01	0.02 ± 0.02	0.004	**0.039**	0.02 ± 0.02	0.02 ± 0.02	0.474
c16	0.02 ± 0.03	0.01 ± 0.01	0.032	0.115	0.01 ± 0.02	0.01 ± 0.01	0.342
c17	0.02 ± 0.02	0.01 ± 0.01	0.263	0.391	0.01 ± 0.01	0.01 ± 0.02	0.370
c18	0.01 ± 0.01	0.02 ± 0.05	0.456	0.459	0.00 ± 0.01	0.01 ± 0.01	0.139

Supplementary Table S2. Lifetime scores (in seconds) of states in partition k3 and associated statistics

	Recurring rrMDD-patients (i.e., relapse at FU N=11)									Nonrecurring rrMDD-patients (i.e., no relapse at FU; N=17)						
state	Baseline mean ± std			Follow-up mean ± std			p	pFDR		Baseline mean ± std			Follow-up mean ± std			p
c1	28.9	±	22.1	33.6	±	43.5	0.34	0.34		33.7	±	28.5	27.0	±	13.3	0.15
c2	5.7	±	2.3	4.9	±	2.3	0.23	0.34		4.8	±	1.7	4.5	±	2.0	0.32
c3	4.4	±	1.6	5.6	±	1.9	0.01	**0.04**		4.6	±	1.8	4.9	±	1.7	0.17

Supplementary Table S3. Sample characteristics of recurrent patients with and without imaging data

		No MRI data (*n*=24)	MRI data (*n*=11)	
Sex	Women	16	9	p=.447[a]
	Men	8	2	
Age		53.8 (7.48)	50.5 (5.75)	$t(33)=1.33$
IQ		108 (7.4)[#]	108 (8.04)[^]	$t(31)=.165$
Episodes in the past (number)		11.62 (15)	11.40 (18)[^]	U=114
HDRS Baseline		3.3 (2.52)[#]	6.11 (5.49)	U=167

Values indicate the mean and standard deviation.
HDRS, Hamilton Depression Rating Scale
[a] Fisher's exact test
[#]n=23; [^]n=10

Supplementary Figure S1

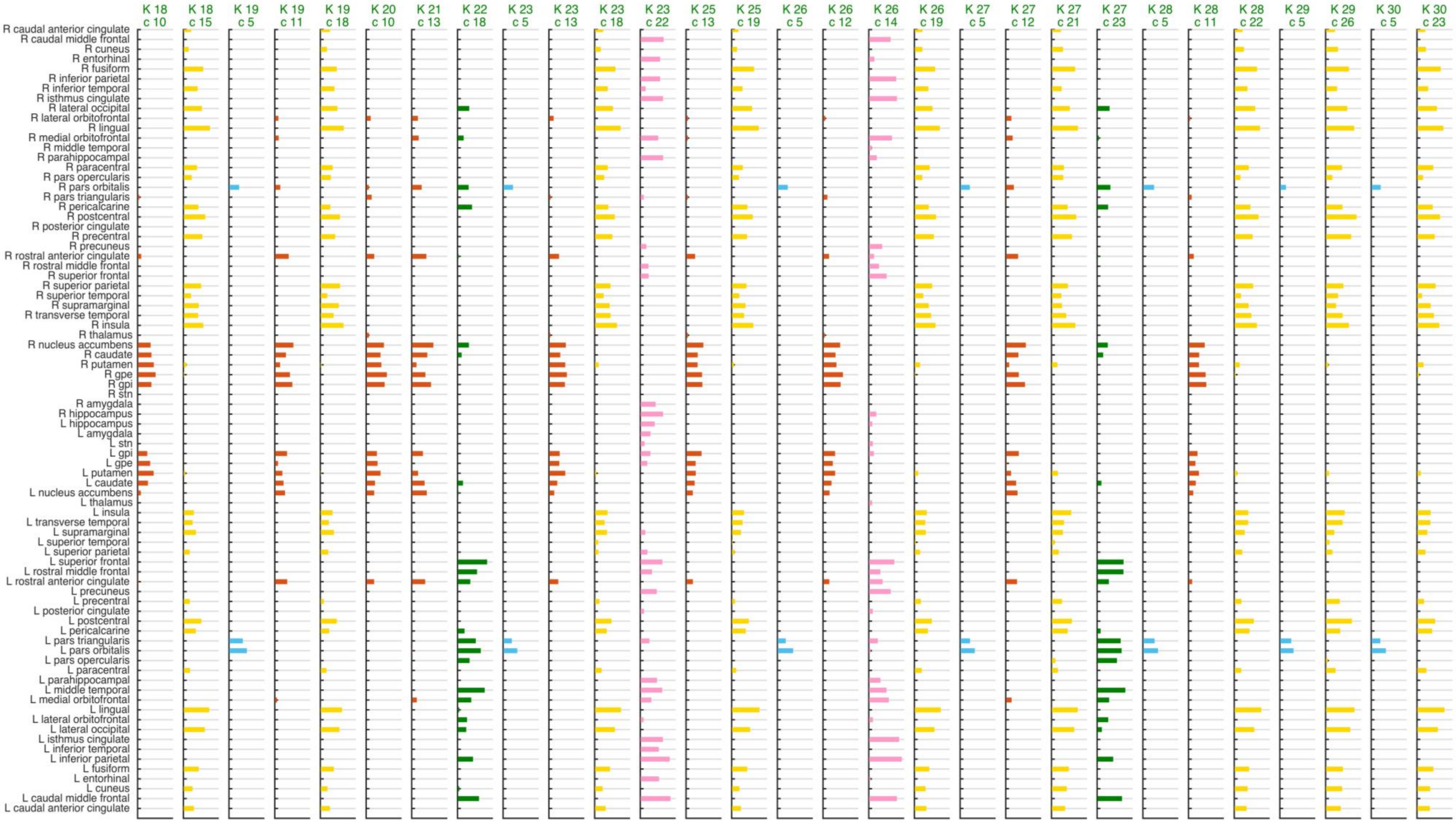

Vector representation of the cluster centroids of PL-states with a significant (p-FDR<0.05) change in their fractional occupancy from baseline (remission) to follow-up (recurrence) when the clustering solution is expanded to 30 clusters. FDR-corrected significance is based on permutation paired t-test (N=11). Each bar plot shows the elements in V_1 representing BOLD signals of brain regions that become coherent and phase-shifted by more than 90° with respect to the BOLD signals in the rest of the brain. K indicates a partition solution into k clusters; c indicates the numbering of the PL-state. Note that for each partition model (k), the PL-states (c) are labelled from 1 to k number of clusters considered in each partition model; as a result, variant forms of the same underlying PL-state do not necessarily have the same label in every partition. Therefore, bar plots of the same color represent similar forms of the underlying PL-state, showing stability of the PL-state across different clustering solutions.
PL = phase-locking.

Supplementary Figure S2.

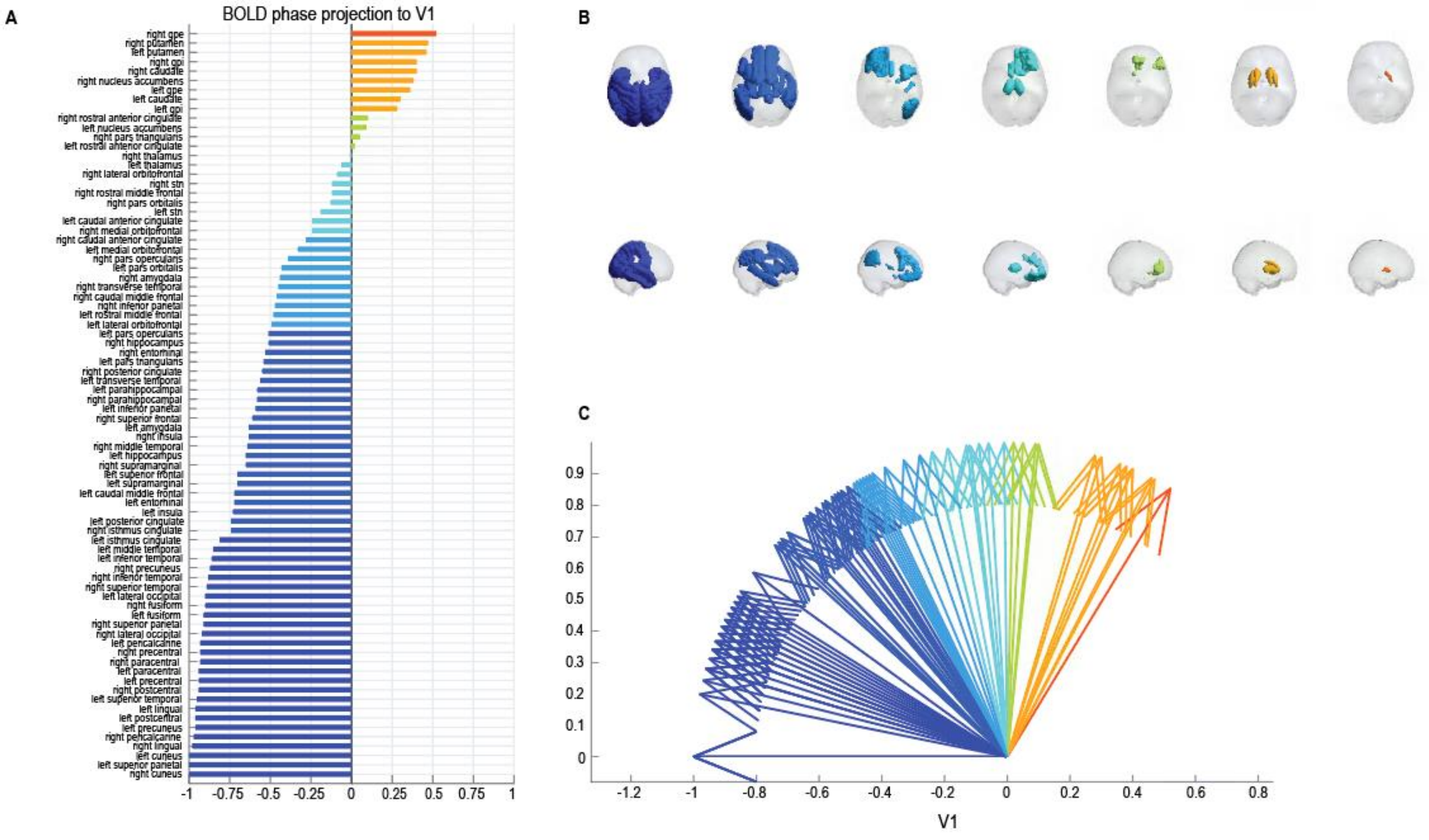

Brain activity during MDD episodes is characterized by a decoupling of the reward system from the rest of the brain. **A** Bar plot shows the elements of the leading eigenvector *V1* of PL-state 10 (k18c10) ordered by strength. Regions of the reward system are depicted in red, orange and green; the BOLD signals of these regions become phase-shifted by more than 90° with respect to the BOLD signals in the rest of the brain (cyan-blue regions). **B** Regions of the same color are rendered in a brain surface. **C** The direction of the arrows indicates the sign of the corresponding element of the Leading eigenvector V_1.

Supplementary Figure S3.

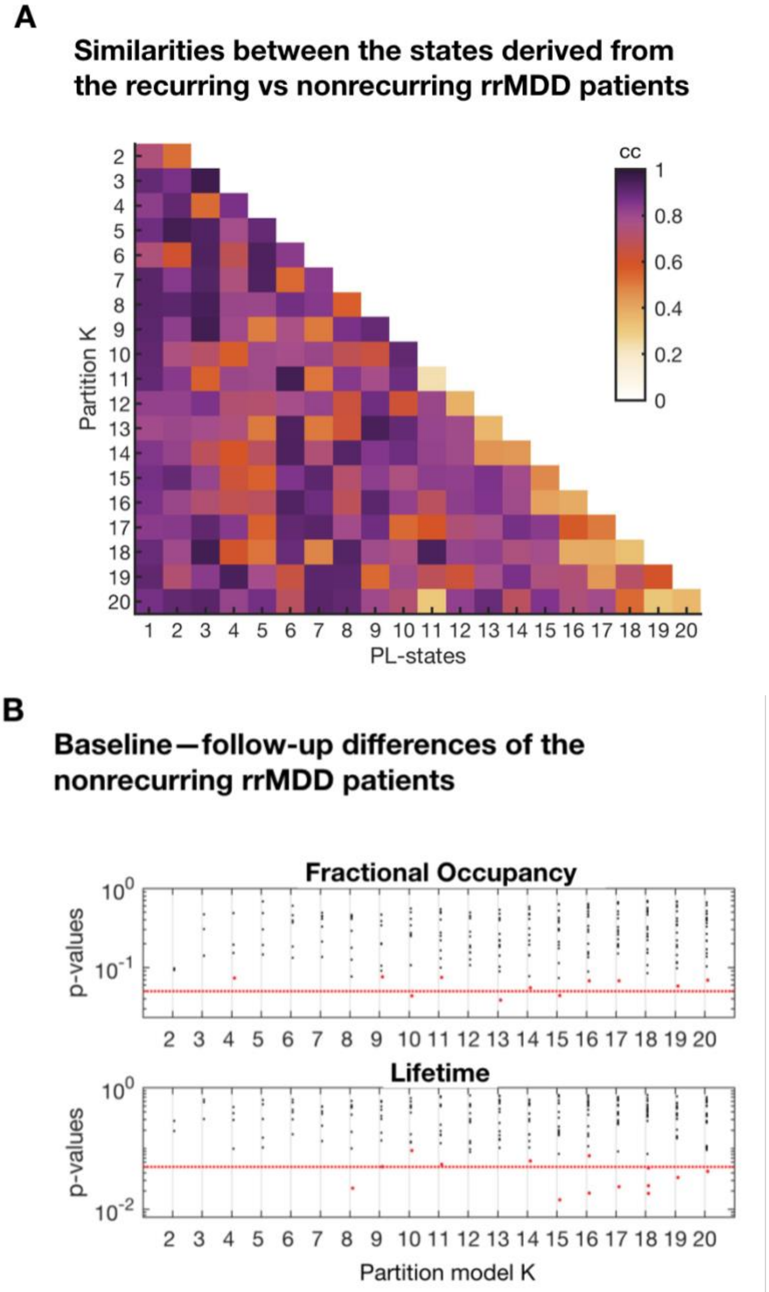

A Similarity matrix shows the correlation coefficients between the PL-states that resulted from clustering the data from the recurring rrMDD patients and the PL-states that resulted from clustering the data from the nonrecurring rrMDD patients. **B** Statistical significance associated with changes in fractional occupancy (top) and lifetime (bottom) between baseline (remission), and follow-up (maintained remission) of the nonrecurring rrMDD patients. Note that contrary to the figures reported in the main text, here, differences between baseline and follow-up scans of the nonrecurring patients were tested on the PL-states defined directly on the data from the nonrecurring patients. Most PL-states do not show significant changes between remission and recurrence (black dots), no PL-states survive FDR corrections (green). PL-states failing to reach the FDR-corrected significance threshold but with $p_{uncorrected}<0.05$ (red dotted line) are indicated in red.
